# An XAI method for convolutional neural networks in self-driving cars

**DOI:** 10.1371/journal.pone.0267282

**Published:** 2022-08-16

**Authors:** Hong-Sik Kim, Inwhee Joe

**Affiliations:** Dept. of Computer and Software, Hanyang University, Seongdong-gu, Seoul, South Korea; Al Mansour University College-Baghdad-Iraq, IRAQ

## Abstract

eXplainable Artificial Intelligence (XAI) is a new trend of machine learning. Machine learning models are used to predict or decide something, and they derive output based on a large volume of data set. Here, the problem is that it is hard to know why such prediction was derived, especially when using deep learning models. It makes the models unreliable in the case of reliability-critical applications. So, it is required to explain how they derived such output. It is a reliability-critical application for self-driving cars because the mistakes made by the computers inside them can lead to critical accidents. So, it is necessary to adopt XAI models in this field. In this paper, we propose an XAI method based on computing and explaining the difference of the output values of the neurons in the last hidden layer of convolutional neural networks. First, we input the original image and some modified images of it. Then we derive output values for each image and compare these values. Then, we introduce the Sensitivity Analysis technique to explain which parts of the original image are needed to distinguish the category. In detail, we divide the image into several parts and fill these parts with shades. First, we compute the influence value on the vector indicating the last hidden layer of the model for each of these parts. Then we draw shades whose darkness is in proportion to the influence values. The experimental results show that our approach for XAI in self-driving cars finds the parts needed to distinguish the category of these images accurately.

## Introduction

eXplainable Artificial Intelligence (XAI) [1] is a field of machine learning. Its goal is to explain how the machine learning models derive outputs. When using XAI to a machine learning model, the model can be more reliable because we can track the process of inference of the model. It is crucial to adopt XAI to self-driving cars because a misunderstanding of the image recognization model adopted for them can lead to deaths. Suppose that we input a picture with the blue sky on the top and the road on the bottom. Then the model can explain that the picture shows a straight road because of the blue sky on the top, not the road on the bottom. Because we need computer vision techniques, we can use Convolutional Neural Networks (CNN) technique for object detection. In short, we need XAI methods for the convolutional neural network of the model used for self-driving cars.

We can classify XAI methods into three major categories. One of them is Sensitivity Analysis (SA) [2], another is Layer-wise Relevance Propagation (LRP) [3], and the other is Feature Importance [4]. We focus on the Sensitivity Analysis method for explaining our convolutional neural network model. It is a method that estimates the influence of each input variable. First, we modify each input variable as a specific value and input the modified input vector to the model. Then we can measure how much the output vector is different from the output vector of the model when the input is the original input vector. So, we can see which elements of the input vector influence the output vector hugely, and so we can see which part of the input makes the model decided rightly or wrongly.

[5] describes five terms about XAI, *understandability*, *comprehensibility*, *interpretability*, *explainability* and *transparency*. Applying this study, We need transparency for our model because one major goal of our study is to prevent accidents that potentially can be made by self-driving cars. Because we use the Sensitivity Analysis method that simulates the layer output of our CNN model, the category of transparency of our XAI model is *simulatability*. Because our model is a CNN model which is not readily interpretable, we should use post-hoc explainability [5]. Our model visualizes the influence of the change of inputs, and we run our XAI model using car-related images such as vehicle and non-vehicle images, the categories that fit the post-hoc explainability of the model are *visual explanation* and *explanations by example*. In addition, [5] describes some goals of XAI, such as *trustworthiess*, *causality* and *transferability*. According to this, the major goal of our XAI model is trustworthiness because our goal is to make a CNN model more trustworthy so that we can prevent potential accidents. Consequently, our XAI model gives transparency to the CNN model by using simulatability, with some example images and visualized explanations about them, to increase the trustworthiness of the CNN model.

There are some previous researches about XAI methods for CNN models. SHAP [6] uses feature importance values for each feature and computes these values by comparing images including and not including this feature. LIME [7] uses an interpretable model which learns with sampled instances from a local area. Grad-CAM [8] uses some counterfactual explanations to change the prediction of CNN, and it can explain any layer, including the last hidden layer. eXplainable CNN (XCNN) [9] uses the network with a heatmap generator of encoder-decoder architecture and creates explanations using the output of this architecture. [10] generates visual explanations using the weighted sum of the feature masks. Two metrics (insertion and deletion) are used for weight computation, using both similarity difference and uniqueness values. [11] make the DCNN (Deep convolutional neural networks) learn from relevant and irrelevant features. It also uses a denoising algorithm and gradient attribution. [12] tries to find the reasons for classification errors using multiple methods and visualizes the last convolutional layer. [13] uses LIME [7] for radar images, with a CNN including various kinds of Keras layers such as ReLU, Batch Normalization, and Flatten. [14] uses a multi-leveled Layer-wise Relevance Propagation (LRP) called Deep Taylor Method for medical images. It experiments on two popular image detection models, Resnet-50 and VGG-16. [15] uses an attribution mask derived from input images, and derives layer visualization map and attribution mask scoring based on the point-wise multiplication between the image and the mask. [16] compares and evaluates LRP with some relevance maps. It compares the average relevance maps and the topo-plots for binary masks for various methods, including LRP-based methods. [17] uses saliency mapping by adding Gaussian noise to the input images. Its data set contains many kinds of galaxy images, and it uses some data augmentation techniques such as random rotations and flips. [18] discovers that natural images are more helpful for providing information about the feature map of CNN than synthetic images. [19] uses a CNN model including Grad-CAM [8] and Gated Recurrent Unit (GRU) for traffic accident anticipation. [20] evaluates how much each type of explanation provides reliable information to people for three different conditions. [21] uses SLRP (a modified version of Layer-wise Relevance Propagation) to find the propagate relevances for each layer of the deep learning models to detect the category of the things in the images, for CNN and RNN. [22] applies Class activation mapping (CAM) to CNN, which uses the weighted sum of image filters of the same size. It uses F-measure and AUC as evaluation metrics. [23] uses some XAI methods such as Grad-CAM and GuidedBP, and its data set contains many mathematical symbols and their combinations. [24] introduces an XAI software, TorchPRISM, and uses the methods such as PCA (principal component analysis) and bilinear interpolation. [25] uses the selected templates corresponding to the feature maps, and represents each category using the set of positive templates.

Some methods [6, 8] use the changes or differences of the input values of a neural network. (refer to the comparison table, Table 1) But our method directly uses these changes and does not include any complex formulas or algorithms. So our method is the most simple compared to these methods among the methods using these changes.

**Table 1:** Method comparison of each related paper.

category of the main method	papers
difference of the input values of neural network	[6, 8]
LIME based methods	[7, 13]
feature (or feature masks)	[10, 11, 15, 22, 25]
LRP-based methods	[14, 16, 21]
others	[9, 12, 17–20, 23, 24]

Table 1 compares each paper from the related works by the category of the method used. Among [6–25], [6, 8] use the difference of the input values of a neural network to generate explanations. We can compare them with our method.

Nowadays, Many electronic things we meet in our life contain machine learning algorithms. There are some kinds of such things that can lead to a critical accident if the decision made by these algorithms are wrong. One of them is self-driving cars. The deep learning models for making decisions usually do not make “explanations” about why they made the decisions, and we do not know the exact value for each neuron from these models. So, the problem is that we cannot know ‘why’ the models made these decisions. In other words, we cannot know which parts of the input image made the model predict like now without XAI techniques. For example, the decision-making model of the self-driving car classified an image as a ‘straight road’ by using the upper part of this image with the blue sky, not using the lower part with the road. In this situation, we can think that the model accuracy is high because it classified the image as a straight road. But when the input image contains only the blue sky, the model can classify it wrongly as ‘straight road’ even if it does not include the road. The solution for this situation is eXplainable Artificial Intelligence (XAI).

Our approach has the four stages below:

- modifying each part of the original input image
- inputting each modified image to the model
- deriving the output
- comparing the output with the output when the input is the original image

In detail, we divide the entire image into many rectangular sub-images with the same width and height. We call each divided sub-image a part, and then we make each modified image by filling each part from the original input images black (RGB 0,0,0). By doing this, We can measure the influence of the change of each part of the input images on the final output.

The main contribution of our method is that we can make meaningfully accurate explanations for the result in a relatively simple way. In the **Related Works** section, there are so many complex methods that try to make an explanation for an image. [6, 8] also generate explanations accurately, but they use Kernel SHAP and complex mathematical operations, and these are not simpler than our method.

## Methods

### Overview

**Fig 1:**
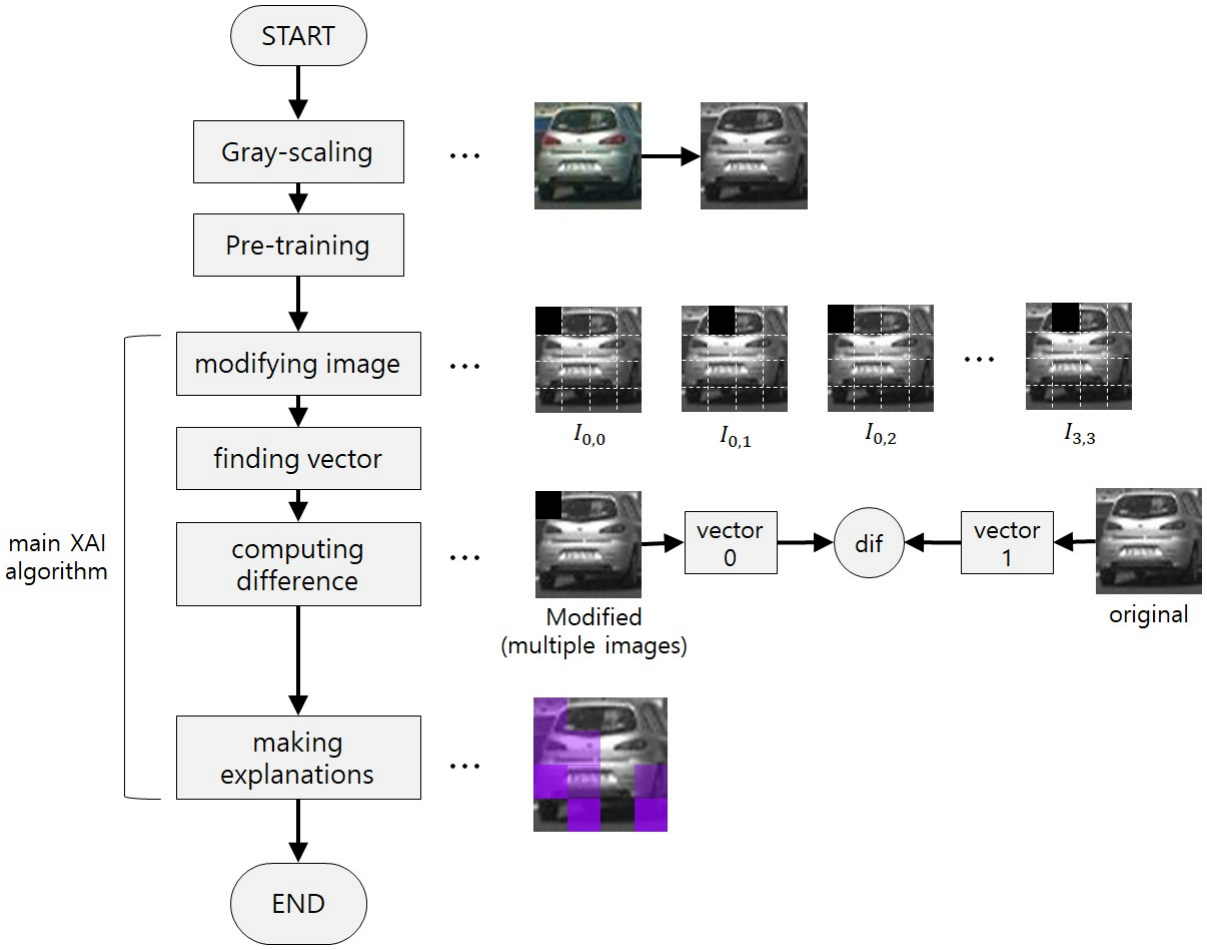
Describes the overall algorithm of our methodology. First, gray-scale images (Gray-scaling), and then pre-train the CNN model (Pre-training), then perform the main XAI algorithm. It contains four stages(steps), that is, **modifying image**, **finding vector**, **computing difference**, and **making explanations**.

A brief description of our methodology is in the flow chart Fig 1. First, we perform **gray-scaling** stage of the images and **pre-training** stage of the CNN model before the main XAI algorithm. Because the pre-training stage is not directly related to XAI, we will explain this later than the main XAI algorithm, in **Experiments and Discussion** section. Next, we go to the main XAI algorithm. Our main XAI algorithm includes four stages(steps). First, **modifying image** is making changes from the original image to explain the changed parts. The algorithm performs it for each part of the image. Second, **finding vector** is inputting the original image and modified images into the network and getting the output vectors of the last hidden layer for each image. Third, **computing difference** is comparing the output vectors of the last hidden layer, when inputting the original image (original output vector) and each modified image (modified output vectors), and then computing the difference between the original output vector and each modified output vector using Euclidean distance. Last, **making explanations** is filling each part of the copied original image in proportion to the difference of the original output vector and each modified output vector, computed in **computing difference**. In practice, inputting and getting the output vector for the original image from **finding vector** earlier than performing **modifying image** has no problem, and in this paper, we performed in this way.

**Fig 2:**
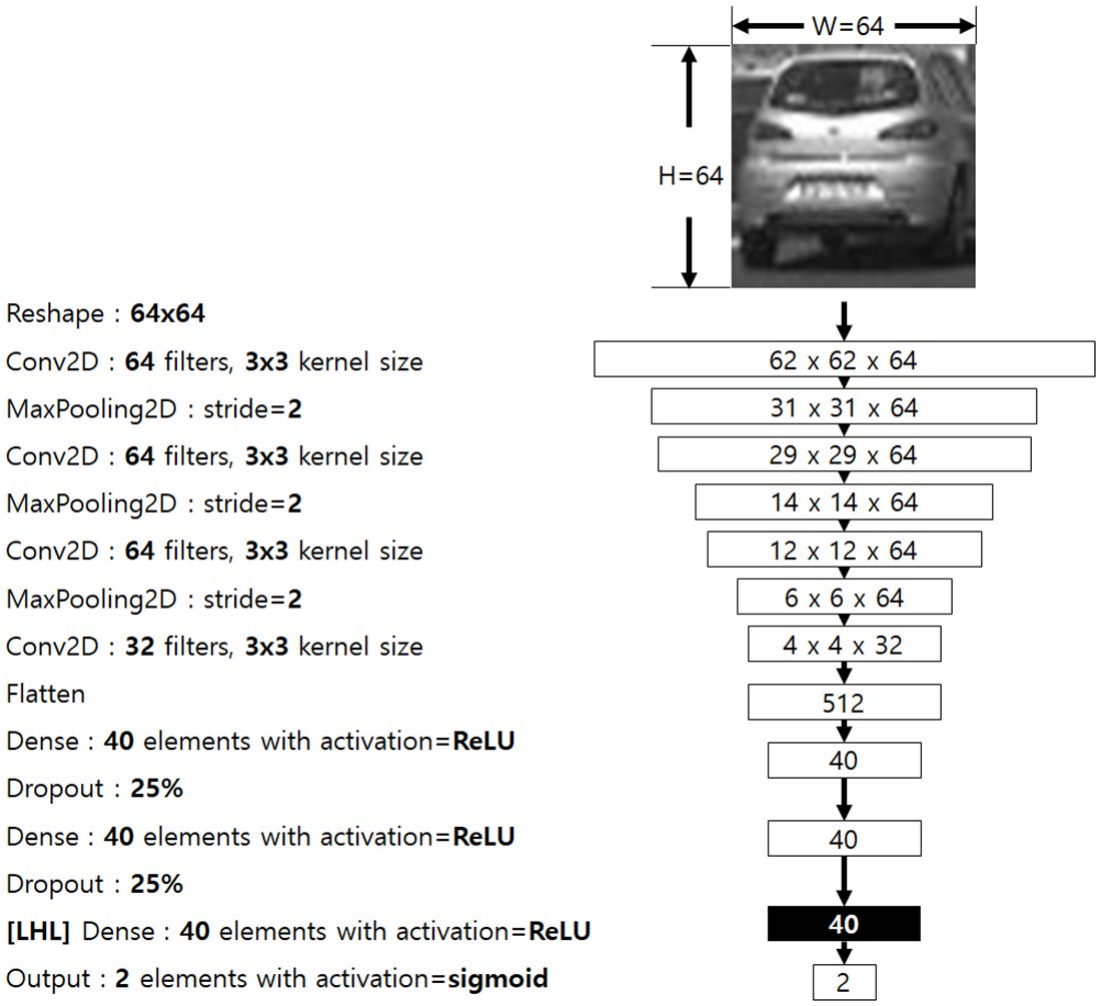
Describes our CNN model. We use the output of the last hidden layer (marked as [LHL]) for estimating the influence of the difference of the input between *I*_*O*_ and each *I*_*n*,*m*_, *n* = 0, …, *N* − 1, *m* = 0, …, *M* − 1.

For **pre-training** stage, we used the CNN model described in Fig 2. We used Tensorflow [26] for model training.

### Main XAI algorithm

**Fig 3:**
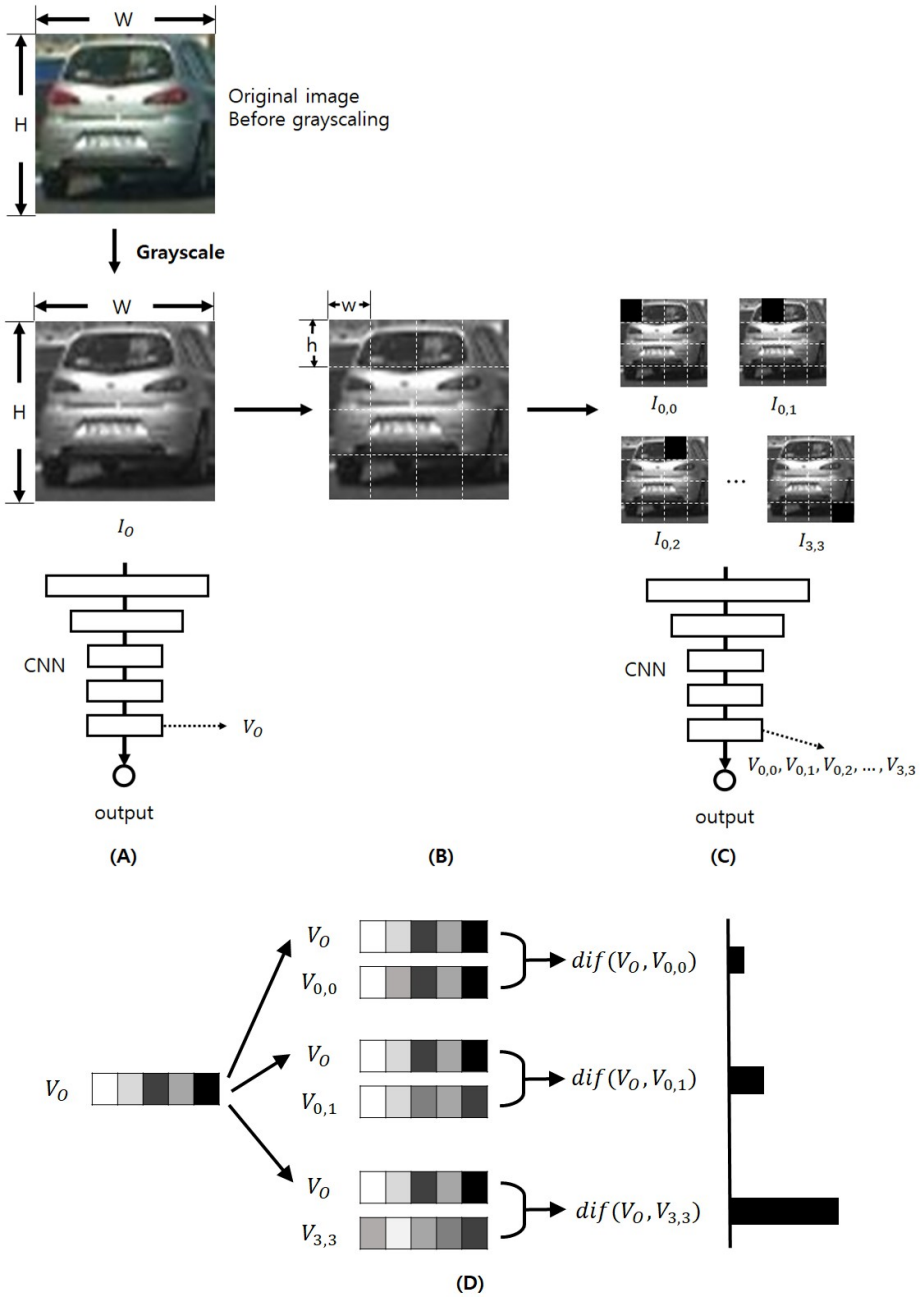
Describes the vector-finding process (step modifying image and finding vector stage) of our method. In **(A)**, we input the original image *I*_*O*_ (VehicleImage/vehicles/Left/image0275.png of [27]) into CNN and get the vector of the last hidden layer, *V*_*O*_. In **(B)**, we divide the original image into *n* × *m* sub-images. In **(C)**, we input each sub-image *I*_0,0_, …, *I*_(*N* − 1), (*M* − 1)_ into the CNN and get the vector of the last hidden layer in the same way. In **(D)**, we compute the difference *dif*(*V*_*O*_, *V*_*n*,*m*_) between *V*_*O*_ and each vector *V*_*n*,*m*_ using Euclidean distance. In the right area of **(D)**, the lengths of horizontal bars mean the values of *V*_*n*,*m*_.

**Fig 4:**
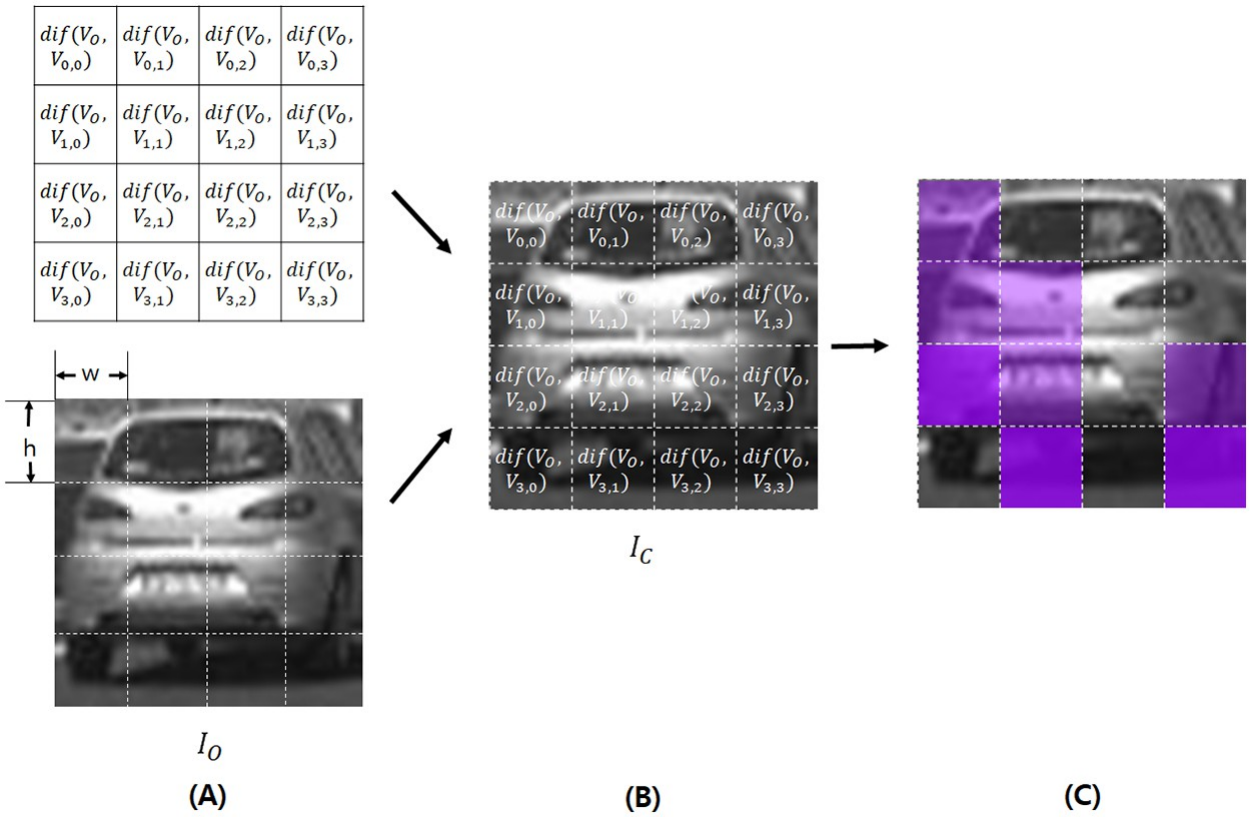
Describes the explanation-deriving process (step computting difference and making explanations) of our method. In **(A)**, we compute the difference dif(*V*_*O*_, *V*_*n*,*m*_) of *V*_*O*_ and each vector *V*_*n*,*m*_, *n* = 0, …, *N* − 1, *m* = 0, …, *M* − 1. In **(B)**, we copy *I*_*O*_ and call it *I*_*C*_. In **(C)**, we fill the resulting image initialized as *I*_*O*_, with gray for each area corresponding to (*n*, *m*)-th sub-image, and the larger the value of dif(*V*_*O*_, *V*_*n*,*m*_), the more clear the purple color for the area. For example, in the figure above, one can see *dif*(*V*_*O*_, *V*_2,0_) is larger than *dif*(*V*_*O*_, *V*_2,1_) because, in **(C)**, the purple color of the cell corresponding to the former is more clear than one corresponding to the latter.

We define each terms as following. *I*_*O*_ means the original image. *W* and *H* means the width and height of *I*_*O*_, respectively. **sub-image** means each divided image from *I*_*O*_. *N* and *M* means the number of sub-image in a column and a row, respectively. *w* and *h* means the width and height of each sub-image, so *N* = *H*/*h* and *M* = *W*/*w*. *V*_*O*_ means the vector of the last hidden layer of CNN when we input *I*_*O*_ into the CNN. *I*_*n*,*m*_, *n* = 0, …, *N* − 1, *m* = 0, …, *M* − 1 means an image whose background is original image, and the area corresponding to (*n*, *m*)-th sub-image is filled with black (RGB 0,0,0). *V*_*n*,*m*_, *n* = 0, …, *N* − 1, *m* = 0, …, *M* − 1 means the vector of the last hidden layer of the CNN when we input *I*_*n*,*m*_, *n* = 0, …, *N* − 1, *m* = 0, …, *M* − 1. *V*_*O*_ can be represented as $\left\{V_{O}^{0},V_{O}^{1},\ldots ,V_{O}^{K-1}\right\}$, where *K* is the number of elements in *V*_*O*_. Like this, *V*_*n*,*m*_ can be represented as $\left\{V_{n,m}^{0},V_{n,m}^{1},\ldots ,V_{n,m}^{K-1}\right\}$, where *K* is the number of elements in *V*_*n*,*m*_, *n* = 0, …, *N* − 1, *m* = 0, …, *M* − 1. Let’s look at Fig 3. First, we convert *I*_*O*_ into gray-scaled image. Then we input *I*_*O*_ to the pre-trained CNN and compute *V*_*O*_ (**(A)**, step **finding vector** for the original image). and then divide *I*_*O*_ into *N* × *M* sub-images, with the height and the width of each image is *h* and *w*, respectively (**(B)**, step **modifying image**). Then, we create and input each *I*_*n*,*m*_, *n* = 0, …, *N* − 1, *m* = 0, …, *M* − 1 and compute *V*_*n*,*m*_, *n* = 0, …, *N* − 1, *m* = 0, …, *M* − 1 in the same way (**(C)**, step **finding vector** for the modified images). From now on, let’s look at Fig 4. We define the difference between *V*_*O*_ and each *V*_*n*,*m*_, *n* = 0, …, *N* − 1, *m* = 0, …, *M* − 1 as dif(*V*_*O*_, *V*_*n*,*m*_), *n* = 0, …, *N* − 1, *m* = 0, …, *M* − 1, as **(1)** (**(A)**, step **computing difference**).

$$\begin{array}{c} \text{dif}\left(V_{O},V_{n,m}\right)=\Sigma_{k}{\left(V_{O}^{k}-V_{n,m}^{k}\right)}^{2},k=0,1,\ldots ,K-1 \end{array}$$ (1)

*K* means the number of elements in the output vector of the last hidden layer of the CNN. Then we copy *I*_*O*_ and call it *I*_*C*_ (**(B)**, preparing the copied original image for step **making explanations**). Then we fill each area of *I*_*C*_ corresponding to *I*_*n*,*m*_, *n* = 0, …, *N* − 1, *m* = 0, …, *M* − 1 with purple (RGB 153,0,255), where the opacity is in the proportion of the value of dif(*V*_*O*_, *V*_*n*,*m*_) (**(C)**, step **making explanations**). Now, we can use *I*_*C*_ to estimate which part of the image influenced the final prediction of the model. We are using the last hidden layer output of CNN instead of the final prediction. There are some reasons for this. First, there are more parameters in the last hidden layer than the final output layer. Second, when the output value of the last hidden layer is influenced more by the difference between the input, the final prediction is also largely influenced (refer to **Experimental Results**). And when the number of neurons in the final output layer is small, the output of this layer can be influenced less. It means that the change of the input values influenced the entire model largely because the output values of the last hidden layer were influenced more.

Our XAI method can be described with Figs 3 and 4 and function **GenerateExplanation** of **Algorithm 1**. In **Algorithm 1**, function grayscale(*img*) receives an image *img*, then performs gray-scaling to *img*, and returns the gray-scaled image. In addition, function predict(*img*, *model*, *i*) receives an input image *img*, a convolutional neural network model *model*, and the layer index *i* = 0, …, *layers* − 1 where *layers* is the number of layers in *model*, including the input layer whose index *i* is 0 and output layer whose index *i* is *layers* − 1. For the layer index *i*, when the output of a layer whose index is *i*_*out*_ and the output is used for the input of the next layer whose index is *i*_*in*_, it is always true that *i*_*out*_ < *i*_*in*_. function Fill(*img*, *y*_0_, *y*_1_, *x*_0_, *x*_1_, *opacity*, *explan*) receives original image *img* and integer values *y*_0_, *y*_1_, *x*_0_, *x*_1_, then copies *img* and fills the square area of copied image with black (RGB 0,0,0) (when *explan* = False) or purple (RGB 153,0,255) (when *explan* = True) with opacity *opacity* whose range is 0.0 to 1.0 (when *explan* = False) or 0.75 (when *explan* = True), where the horizontal and vertical range of the area are from *x*_0_ to *x*_1_ (*x*_0_ < *x*_1_) and from *y*_0_ to *y*_1_ (*y*_0_ < *y*_1_), respectively.

**Algorithm 1** Generating an explanation image (like **(C)** in Fig 4) for an image

**function** Fill(*img*, *y*_0_, *y*_1_, *x*_0_, *x*_1_, *opacity*, *explan*)

 *newImg* ← copy(*img*)

 **for** each *y* = *y*_0_, …, *y*_1_ − 1 **do**

  **for** each *x* = *x*_0_, …, *x*_1_ − 1 **do**

   *pixel*_*R*_, *pixel*_*G*_, *pixel*_*B*_ ← R, G, B value of the (*y*, *x*)-th pixel, respectively

   **if** *explan* = True **do**

    *newPixel*_*R*_ ← 0.6*opacity + *pixel*_*R*_ × (1.0 − *opacity*)

    *newPixel*_*G*_ ← *pixel*_*G*_ × (1.0 − *opacity*)

    *newPixel*_*B*_ ← 1.0*opacity + *pixel*_*B*_ × (1.0 − *opacity*)

   **else do**

    *newPixel*_*R*_ ← *pixel*_*R*_ × (1.0 − *opacity*)

    *newPixel*_*G*_ ← *pixel*_*G*_ × (1.0 − *opacity*)

    *newPixel*_*B*_ ← *pixel*_*B*_ × (1.0 − *opacity*)

   **end if**

   R, G, B value of the (*y*, *x*)-th pixel of *newImg* ← *newPixel*_*R*_, *newPixel*_*G*_, *newPixel*_*B*_, respectively

  **end for**

 **end for**

**return** *newImg*

**end function**

**function** GenerateExplanation(*I*_*O*_, *W*, *H*, *N*, *M*, *model*)

 *I*_*O*_ ← grayscale(*I*_*O*_)

 *explanation* ← copy(*I*_*O*_)

 *layers* ← the number of layers in *model*, including input and output layers

 *V*_*O*_ ← predict(*I*_*O*_, *model*, *layers*-2)

 *w* ← *W*/*M*, *h* ← *H*/*N*

 **for** each *n* = 0, …, *N* − 1 **do**

  **for** each *m* = 0, …, *M* − 1 **do**

   *I*_*n*,*m*_ ← Fill(*I*_*O*_, *nh*, (*n* + 1)*h*, *mw*, (*m* + 1)*w*, 1.0, False)

   *V*_*n*,*m*_ ← predict(*I*_*n*,*m*_, *model*, *layers*-2)

   *D*_*n*,*m*_ ← dif(*V*_*O*_, *V*_*n*,*m*_)

  **end for**

 **end for**

 *max*_*D*_ ← max(*D*_*n*,*m*_, *n* = 0, …, *N* − 1, *m* = 0, …, *M* − 1)

 **for** each *n* = 0, …, *N* − 1 **do**

  **for** each *m* = 0, …, *M* − 1 **do**

   *D* ← 0.75*D_*n*,*m*_/*max*_*D*_

   *explanation*← Fill(*explanation*, *nh*, (*n* + 1)*h*, *mw*, (*m* + 1)*w*, *D*, True)

  **end for**

 **end for**

 **return** *explanation*

**end function**

## Experiments and discussion

The name of the steps of our method (**pre-training**, **modifying image**, **finding vector**, **computing difference** and **making explanations**) can be referred in this section including subsections, and these names are from section **Overview**.

We used the test images and pre-trained CNN model of the step **Pre-training** for the experiment. It means we input the test images into the pre-trained CNN and got the result. We set *W* = 64, *H* = 64, *M* = 8 and *N* = 8 for our experiment, and so the value of *w* and *h* is both 8. The programming language we used is Python 3.7, and Operating System is Windows 10. You can download the dataset used for this experiment from [27, 28], and Python code used for this experiment from https://github.com/WannaBeSuperteur/2020/tree/master/AI/CAR_test_202102 (Vehicle vs. Non-vehicle [27]), https://github.com/WannaBeSuperteur/2020/tree/master/AI/SIGN_test_202109 (traffic signs [28]), https://github.com/WannaBeSuperteur/2020/tree/master/AI/SIGN_fewclasses_test_202109 (traffic signs [28] with few classes) and https://github.com/WannaBeSuperteur/2020/tree/master/AI/SIGN_speedlimit_test_202110 (traffic signs [28] (only speed limit signs).

### Pre-training

We pre-trained the CNN with four image datasets downloaded from [27, 28]. Table 2 describes detailed dataset information. For example, The dataset named “Vehicle vs. Non-vehicle” [27] contains 7,325 images in total (3,900 non-vehicle images and 3,425 vehicle images). We split these images into training images and test images, where the proportion of images for training and test is 0.8 and 0.2, respectively. So we have 3,120 training images and 780 test images for the non-vehicle category. Also, we have 2,740 training images and 685 test images for the vehicle category.

**Table 2:** Information of each dataset used.

dataset name	images (for train, for test)	catgs	MSE	ACC
Vehicle vs. Non-vehicle [27]	7,325 (5,860, 1,465)	2	0.0257	0.9700
traffic signs [28]	39,209 (31,367, 7,842)	43	0.0036	0.9086
traffic signs [28] with few classes	39,209 (31,367, 7,842)	7	0.0013	0.9945
traffic signs [28] (only speed limit signs)	12,780 (10,224, 2,556)	8	0.0164	0.9198

Table 2 shows the number of images for training and test, the number of categories of the images (catgs), MSE(mean-squared error), and the accuracy of each pre-trained CNN model in Fig 2, using the dataset (MSE and ACC, respectively).

For each dataset, we trained the CNN model with all the images for training and then evaluated with Mean Square Error (MSE) and accuracy. (stage **pre-training**) The final output contains N elements which indicate each category, where N is the number of categories in the dataset. We measured the accuracy using (**correct count**) / (total number of images). **correct count** is defined as the number of images whose index of the largest element in the final output vector for the prediction is the same as for the ground truth.

The dataset “traffic signs with few classes” used the traffic signs dataset [28], but we reduced the number of classes. We marked each image as below. “A: B” means that we marked the image as A if whose original class is one of B.

- “class 0”: 0, 1, 2, …, or 8
- “class 1”: 9, 10, 16, 41 or 42
- “class 2”: 11, 19, 20 or 21
- “class 3”: 12, 13, 15 or 32
- “class 4”: 14, 17 or 18
- “class 5”: 22, 23, 24, …, or 31
- “class 6”: 33, 34, 35, …, or 40

The dataset “traffic signs [28] (only speed limit signs)” also used the traffic signs dataset [28], but we only used ‘speed limit’ sign images whose original classes are 0, 1, 2, 3, 4, 5, 7, and 8. We marked each image as class 0, 1, 2, 3, 4, 5, 6, and 7 to the images whose original class is 0, 1, 2, 3, 4, 5, 7, and 8, respectively.

### Experiment for XAI algorithm

**Fig 5:**
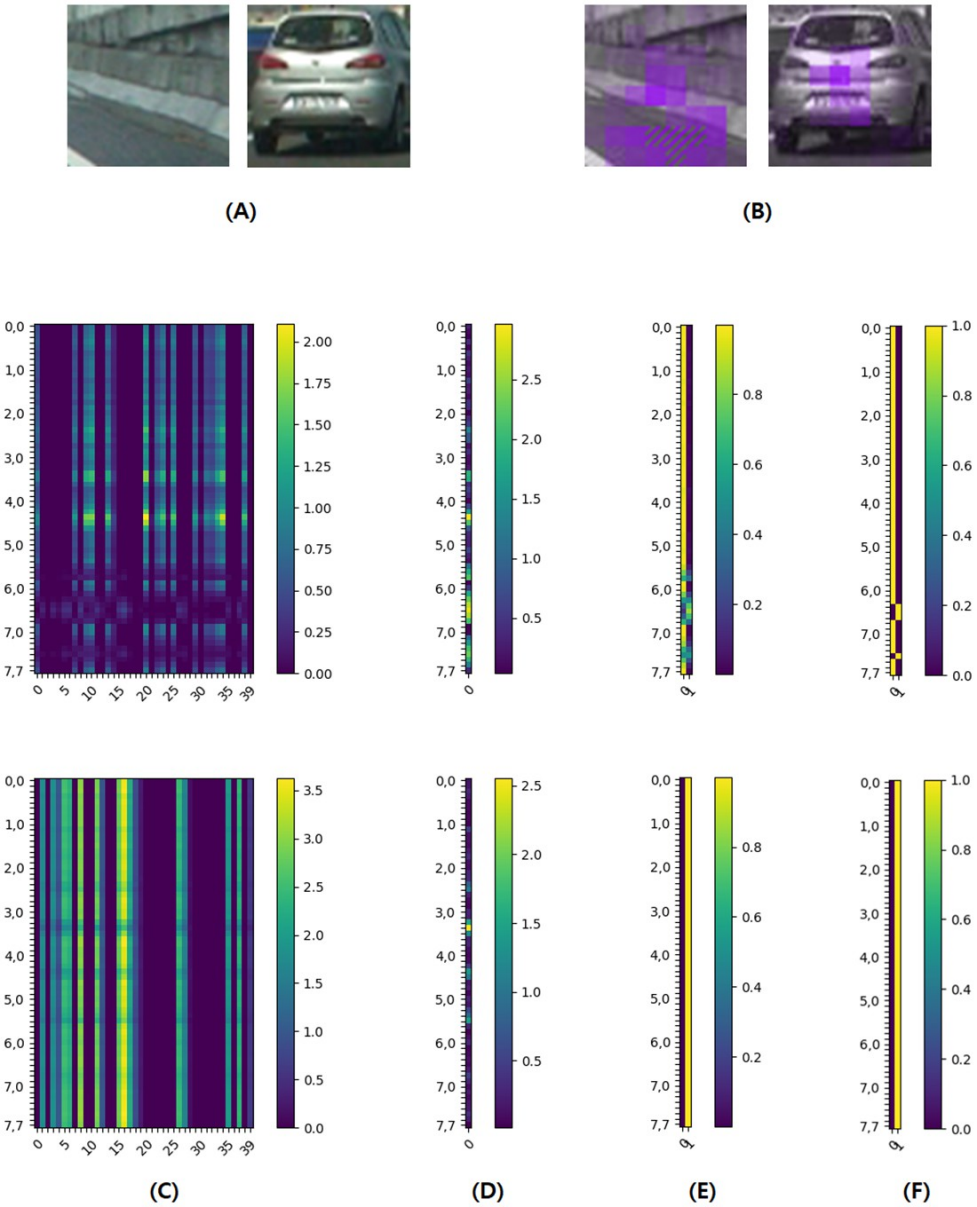
Describes an example of our CNN-explaining for an image. **(A)** and **(B)** describes the result of step **making explanations** and **(C)**-**(F)** describes the result of step **computing difference**. **(A)** is the original images *I*_*O*_ and **(B)** is *I*_*C*_’s. In **(B)**, when the final output predictions of *I*_*n*,*m*_’s are different from *I*_*O*_’s, we marked the corresponding area as upward-right lines. **(C)** is the visualization of $|V_{O}^{k}-V_{n,m}^{k}|,k=0,1,\ldots ,K-1,m=0,1,\ldots ,M-1,n=0,1,\ldots ,N-1$ with *K* = 40. Each row of **(C)** means $|V_{O}^{k}-V_{n,m}^{k}|,k=0,1,\ldots ,K-1$ for each fixed *n* and *m*. **(D)** is dif(*V*_*O*_, *V*_*n*,*m*_) for each fixed *n* and *m*. **(E)** is the visualization of the final output for each fixed *n* and *m*, and **(F)** is the binary-ized values for **(E)**. For all the images **(C)**-**(F)**, the lighter the color of each cell, the larger the corresponding value. The left (non-vehicle, from VehicleImage/non-vehicles/Right/image0825.png of [27]) and right (vehicle, from VehicleImage/vehicles/Left/image0275.png of [27]) images in **(A)** and **(B)** is corresponding to the upper and lower images in **(C)**-**(F)**, respectively.

**Fig 6:**
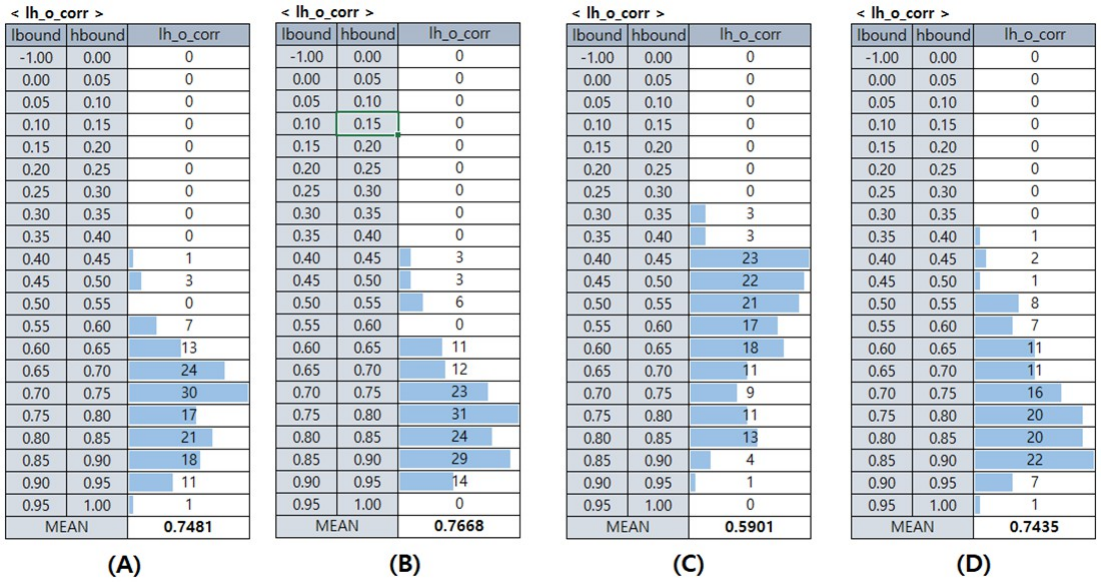
Describes the distribution of *lh*_*o*_*corr* (the result of step computing difference) for all the test images. the number in the column *lh*_*o*_*corr* means the number of cases with *lh*_*o*_*corr* value between *lbound* and *hbound*. **(A)**, **(B)**, **(C)**, **(D)** means the *lh*_*o*_*corr* values of the dataset “Vehicle vs. Non-vehicle”, “traffic signs”, “traffic signs with few classes” and “traffic signs (only speed limit signs)”, respectively. There is no case that *lh*_*o*_*corr* is less than 0 for any dataset, and the average of *lh*_*o*_*corr* is about 0.7.

We created the image explaining our experimental result using matplotlib [29]. Fig 5 describes our experimental results for a test image, related to the step **computing difference** and **making explanations**. We can see the larger the difference $\text{dif}(V_{O}^{k},V_{n,m}^{k}),m=0,1,\ldots ,M-1,n=0,1,\ldots ,N-1$, the larger the difference of the final output vector. For this image, when (*n*, *m*) is (6, 3), (6, 4), (6, 5) or (7, 4), the final output is different from the final output for *I*_*O*_, and the differences $\text{dif}(V_{O}^{k},V_{6,3}^{k})$, $\text{dif}(V_{O}^{k},V_{6,4}^{k})$, $\text{dif}(V_{O}^{k},V_{6,5}^{k})$ and $\text{dif}(V_{O}^{k},V_{7,4}^{k})$ are very large. Fig 6 describes the distribution and average value of *lh*_*o*_*corr* for each dataset. Table 3 describes the correlation coefficients between each pair of two variables we think meaningful. The description of each variable is as Table 4. Fig 6 and Table 3 are related to the step **computing difference**.

**Table 3:** Correlation coefficient for each pair of two variables.

**(A)**		*difCells*	*avgRank*	*maxRank*	*samMax*	*difMin*	*sm* − *dm*	*O*/*d*	*m* − 2*n*	*lhocr*
	*difCells*			0.678	-0.484	0.729	0.523	0.920	-0.908	0.073
	*avgRank*			0.678	-0.484	0.729	0.523	0.920	-0.908	0.073
	*maxRank*	0.678	0.678		-0.632			0.635	-0.635	-0.079
	*samMax*	-0.484	-0.484	-0.632		-0.189	0.589	-0.390	0.401	0.223
	*difMin*	-0.729	-0.729		-0.189		-0.905	-0.870	0.870	-0.285
	*sm* − *dm*	0.523	0.523		0.589	-0.905		0.714	-0.730	0.306
	*O*/*d*	0.920	0.920	0.635	-0.390	-0.870	0.714		-0.984	0.135
	*m* − 2*n*	-0.908	-0.908	-0.635	0.401	0.870	-0.730	-0.984		-0.082
	*lhocr*	0.073	0.073	-0.079	0.223	-0.285	0.306	0.135	-0.082	
**(B)**		*difCells*	*avgRank*	*maxRank*	*samMax*	*difMin*	*sm* − *dm*	*O*/*d*	*m* − 2*n*	*lhocr*
	*difCells*		0.861	0.470	-0.318	-0.454	0.104	0.699	-0.815	0.356
	*avgRank*	0.861		0.538	-0.278	-0.324	0.024	0.499	-0.606	0.274
	*maxRank*	0.470	0.538		-0.081	-0.222	0.096	0.465	-0.345	0.493
	*samMax*	-0.318	-0.278	-0.081		0.495	0.497	-0.269	0.253	-0.128
	*difMin*	-0.454	-0.324	-0.222	0.495		-0.507	-0.528	0.432	-0.369
	*sm* − *dm*	0.104	0.024	0.096	0.497	-0.507		0.218	-0.158	0.153
	*O*/*d*	0.699	0.499	0.465	-0.269	-0.528	0.218		-0.784	0.599
	*m* − 2*n*	-0.815	-0.606	-0.345	0.253	0.432	-0.158	-0.784		-0.337
	*lhocr*	0.356	0.274	0.493	-0.128	-0.369	0.153	0.599	-0.337	
**(C)**		*difCells*	*avgRank*	*maxRank*	*samMax*	*difMin*	*sm* − *dm*	*O*/*d*	*m* − 2*n*	*lhocr*
	*difCells*		0.955	0.578	-0.162	-0.397	0.144	0.890	-0.826	0.377
	*avgRank*	0.955		0.677	-0.227	-0.362	0.062	0.811	-0.740	0.389
	*maxRank*	0.578	0.677		-0.211	-0.195	-0.088	0.574	-0.457	0.664
	*samMax*	-0.162	-0.227	-0.211		0.038	0.700	-0.135	0.113	-0.003
	*difMin*	-0.397	-0.362	-0.195	0.038		-0.687	-0.477	0.343	-0.307
	*sm* − *dm*	0.144	0.062	-0.088	0.700	-0.687		0.222	-0.150	0.179
	*O*/*d*	0.890	0.811	0.574	-0.135	-0.477	0.222		-0.925	0.487
	*m* − 2*n*	-0.826	-0.740	-0.457	0.113	0.343	-0.150	-0.925		-0.307
	*lhocr*	0.377	0.389	0.664	-0.003	-0.307	0.179	0.487	-0.307	
**(D)**		*difCells*	*avgRank*	*maxRank*	*samMax*	*difMin*	*sm* − *dm*	*O*/*d*	*m* − 2*n*	*lhocr*
	*difCells*		0.936	0.374	-0.069	-0.497	0.149	0.865	-0.820	0.247
	*avgRank*	0.936		0.617	0.032	-0.531	0.270	0.795	-0.732	0.357
	*maxRank*	0.374	0.617		0.245	-0.412	0.512	0.295	-0.165	0.550
	*samMax*	-0.069	0.032	0.245		-0.185	0.933	-0.015	0.074	0.008
	*difMin*	-0.497	-0.531	-0.412	-0.185		-0.527	-0.479	0.383	-0.226
	*sm* − *dm*	0.149	0.270	0.512	0.933	-0.527		0.198	-0.087	0.133
	*O*/*d*	0.865	0.795	0.295	-0.015	-0.479	0.198		-0.900	0.302
	*m* − 2*n*	-0.820	-0.732	-0.165	0.074	0.383	-0.087	-0.900		-0.174
	*lhocr*	0.247	0.357	0.550	0.008	-0.226	0.133	0.302	-0.174	

Table 3 describes the correlation coefficient between each pair of two variables, according to *O*_*argmax*_, derived using the step **computing difference**. The definition of each variable is from Table 2. Like Fig 6(A)–6(D) means the *lh*_*o*_*corr* values of the dataset “Vehicle vs. Non-vehicle”, “traffic signs”, “traffic signs with few classes” and “traffic signs (only speed limit signs)”, respectively. For (*difMin*, *maxRank*) and (*difMin*, *smax* − *dmin*) of **(A)**, we cannot compute the correlation coefficients because of the records whose *difMin* and *smax* − *dmin* value is available always have the *maxRank* value as 2. *sm* − *dm*, *O*/*d*, *m* − 2*n* and *lhocr* means *smax* − *dmin*, *O*_*dif*/*dif*_*n*,*m*_, *max* − 2*nd* and *lh*_*o*_*corr*, respectively.

**Table 4:** Meanings of variables for experimental results.

term	description
*O*_*argmax*_	the index (starts with 0) of the largest value from *V*_*O*_
*n*, *m*_*argmax*_	the index (starts with 0) of the largest value from *V*_*n*,*m*_
*difCells*	the number of (*n*, *m*)’s that *O*_*argmax*_ is different from *n*, *m*_*argmax*_
*Rank*	the rank of the value $V_{n,m}^{O_{argmax}}$, among all the values from *V*_*n*,*m*_
*avgRank*	the average value of *Rank* for all the (*n*, *m*)’s
*maxRank*	the maximum value of *Rank* for all the (*n*, *m*)’s
*dif*_*n*,*m*_	the average value of dif(*V*_*O*_, *V*_*n*,*m*_)
*O*_*O*_	the final output vector when we input *I*_*O*_ to the CNN
*O*_*n*,*m*_	the final output vector when we input *I*_*n*,*m*_ to the CNN
*O*_*dif*	the average value of dif(*O*_*O*_, *O*_*n*,*m*_)
*samMax*	the maximum value of *dif*_*n*,*m*_ when *Rank* = 1, among all the *n*’s and *m*’s
*difMin*	the minimum value of *dif*_*n*,*m*_ when *Rank* > 1, among all the *n*’s and *m*’s
*smax* − *dmin*	the value of *samMax* − *difMin*
*O*_*dif*/*dif*_*n*,*m*_	the ratio between *O*_*dif* and *dif*_*n*,*m*_
*max*_*n*,*m*_	the maximum value among all the values from *O*_*n*,*m*_
2*nd*_*n*,*m*_	the second maximum value among all the values from *O*_*n*,*m*_
*max* − 2*nd*	average value of *max*_*n*,*m*_ − 2*nd*_*n*,*m*_, for all the *n*’s and *m*’s
*lh*_*o*_*corr*	correlation coefficient of *O*_*dif* and *dif*_*n*,*m*_

Table 4 shows the meaning of each variable. The range of *n* and *m* is always *n* = 0, …, *N* − 1 and *m* = 0, …, *M* − 1 respectively.

### Discussion

First, we show the examples of our result based on the dataset named “Vehicle vs. Non-vehicle”. From Fig 5, we can see that for the non-vehicle image (left image of **(A)** and **(B)**), the lower parts of the image influence much more than the upper ones. Because we distinguish vehicle images from non-vehicle images using whether there are wheels at the bottom of the object, our model can use the bottom part of the image to decide whether it is a vehicle or not. So, the result in the left image of **(B)** says our XAI model can bring meaningful results. For the vehicle image (right image of **(A)** and **(B)**), we can see that the middle and lower parts of the image influence more than the other parts, because the model we designed usually filled upper parts as less clear purple and lower parts as more clear purple. Specifically, the background parts in the top and left of the image influenced much less than the middle and lower parts. Unlike the non-vehicle images, the center part largely influenced the final prediction and the last hidden layer output for the vehicle images. The reason is that we usually distinguish vehicles from non-vehicles using the shape and color of their body. That is, we can recognize it as an object other than a vehicle if the shape and color do not match the image of vehicles.

From Fig 6, we can see that for most of the images, the correlation between the average changes of the final output vector (*O*_*dif*) and the last hidden layer output vector (*dif*_*n*,*m*_) is positive enough. There is no image that they have a negative correlation. Among the four datasets we used, the maximum value of the correlation coefficient is between 0.95 and 1.0, from “Vehicle vs. Non-vehicle” and “traffic signs (only speed limit signs)”. The minimum value is between 0.3 and 0.35, from “traffic signs with few classes”. The dataset with the highest *lh*_*o*_*corr* is “traffic signs” (0.7668), and the lowest is “traffic signs with few classes” (0.5901).

From Table 3, we can see that there are positive correlations among *difCells*, *avgRank*, *maxRank*, *smax* − *dmin* and *O*_*dif*_/*dif*_*n*,*m*_ (group 1), and between *difMin* and *max* − 2*nd* (group 2), and negative ones among the two groups. For the dataset “Vehicle vs. Non-vehicle”, because there are only 2 classes(non-vehicle and vehicle) so *Rank* can have only 2 values (1 or 2), *difCells* have a linear correlation with *avgRank*. It is trivial that *avgRank* and *maxRank* have positive correlations. *O*_*dif*_/*dif*_*n*,*m*_ have positive correlation with *difCells*, because when dif(*O*_*O*_, *O*_*n*,*m*_) is larger, the final prediction can change easier, it indicates larger *difCells*. We define the two cases of images here: **Case 1** is the images whose final prediction of CNN can be changed by small changes on it, and **Case 2** is the opposite. Because *smax* − *dmin* is large when *samMax* is large and *difMin* is small, and the value of *difMin* for **Case 2** is larger than **Case 1**, and the value of *Rank* is higher than **Case 2** than **Case 1**, *maxRank* and *smax* − *dmin* have positive correlations, and they have negative ones with *difMin*. Because for **Case 1** images with larger *difMin* values, the two elements of the final prediction vector of them have larger difference, and it indicates they have larger *max* − 2*nd* values, *difMin* and *max* − 2*nd* have positive correlation. *lh*_*o*_*corr* have a weak positive correlation with the variables from group 1, because the images with meaningful (just enough, not means large) dif(*V*_*O*_, *V*_*n*,*m*_) makes *lh*_*o*_*corr* larger, and **Case 1** images have large dif(*V*_*O*_, *V*_*n*,*m*_). The appearance of the correlation coefficients between every two variables, as mentioned above, has consistency among all the datasets used for this experiment.

**Fig 7:**
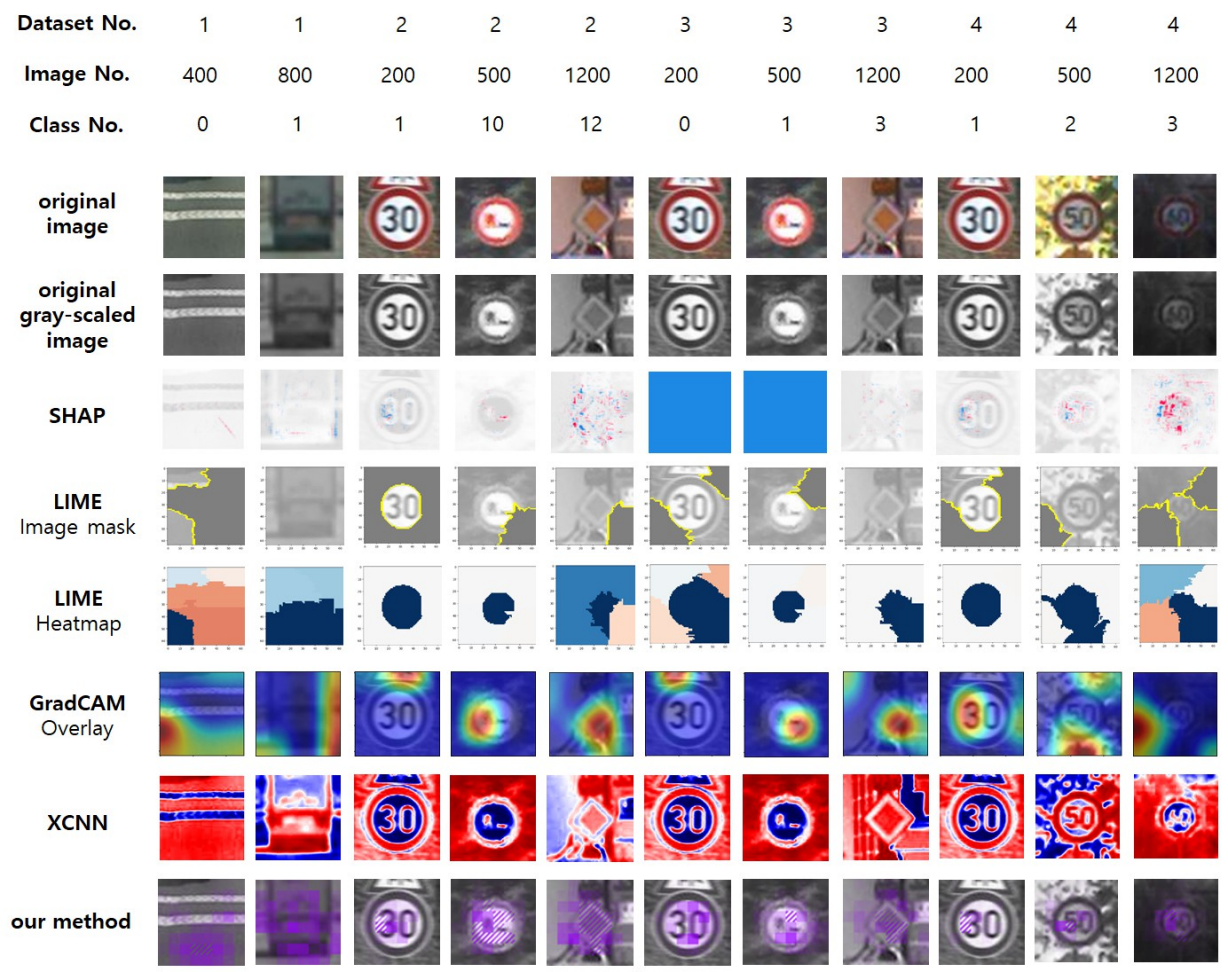
Describes the comparison of our method with some other methods. They include SHAP [6], LIME [7], Grad-CAM [8] and eXplainable CNN (XCNN) [9]. “Dataset No.” is 1, 2, 3, and 4 for “Vehicle vs. Non-vehicle”, “traffic signs”, “traffic signs with few classes” and “traffic signs (only speed limit signs)”, respectively, “Class No.” is the ground truth of the class of each image, and “Image No.” is the index of each image among all the images-for-test from the dataset which includes this image.

Fig 7 describes the comparison of our method with SHAP [6], LIME [7], Grad-CAM [8] and eXplainable CNN (XCNN) [9]. The methodologies used in these papers are described in **Related Works** section. We implemented and executed each algorithm with Python, and the codes for them include some code snippets from [30–33], respectively. One can see our code for this comparison in [34]. The core point of our model, which any of these methods to compare don’t have, is that one can see which part of the image influences the final prediction and the output of LHL directly, with the fill color for each part of the image. The highlighted parts in the resulting images of SHAP, LIME, Grad-CAM, XCNN, and our method are all different for most cases. Another core point of our model is that except for the cases where any modification cannot influence the final output prediction, our model does not fail for any image, likely SHAP and unlikely LIME and Grad-CAM. We can make the final explanation visually better by applying other colors to fill for the explanation made by the model. Also, by increasing the value of *M* and *N*, we can describe more detailed explanations.

SHAP, image mask of LIME, Grad-CAM, and our method show the original images with explanations so that one can find which part of the image influenced the final prediction easily. Among these methods, SHAP explains in detail and shows the parts which have positive influences increasing the probability for a class, and negative influences decreasing it, with different colors. But it fails for some images such as 6th and 7th left images whose SHAP results are ‘completely blue’. LIME seems to be failed for both image mask and heatmap because the size of the original image is quite small (64x64). Because LIME uses segmentation for pixels, it is not good for small-size images, so the result is quite bad. In addition, there are some images (2nd and 8th left image) that LIME failed to make explanations. Grad-CAM highlights the area more smoothly than other methods and seems successful, but in the 3rd left image, it shows a failure. In this image, it highlights not the circular sign with ‘30’, but the triangle sign above it. XCNN seems to just show the ‘texture’ of the images, and it is not enough explanation for the images.

For the images whose Dataset No. is 4 (dataset name: traffic signs (only speed limit signs), the rightmost three images), the classification result is decided by the number(s) on the left of the rightmost ‘0’. That is because it is decided by the number inside the sign, and the rightmost number is always ‘0’. So, these number(s) are the most important things to decide the class of images, and XAI methods should highlight these number(s). For example, if the image contains the speed limit sign with ‘80’, XAI methods should highlight ‘8’ on the left of ‘0’. Considering these images, only the two methods (SHAP and our method) are successful. Grad-CAM is successful for only one image (the 3rd right image) among these three images. Consequently, because SHAP fails for some images as mentioned above, our method shows meaningful XAI performance for car-related things images.

## Conclusion

We created an XAI model to see how the CNN model predicts the class of image and which part of the image influences the final prediction of the CNN model more than other parts. Also, we designed the model to explain the influence of each part on the final prediction. Also, we defined, measured, and analyzed the variables about the details of the XAI model. As from **Discussion** section, the XAI model works well for vehicle images, non-vehicle images, and other car-related datasets.

Our research can contribute to the CNNs used for self-driving cars by providing a simple and intuitive XAI system. The main contribution is that we proposed a simplified method for the XAI process for these two main contributions below, for self-driving cars, and it is also the main difference between our method and the previous methods.

- First, our research can detect and help analyze the prediction pattern of the CNN model so it can measure and ensure the credibility of the CNN model. So we can help to improve the CNN model. For example, we can run our model with images with both an object and the background. In this experiment, if the influence of the part corresponding to the object is much more than the part corresponding to the background, we can see that the CNN model is credible.
- Second, when the self-driving car causes an accident, we can analyze how and why the CNN model failed to predict correctly. For example, suppose that the model predicted an object in an image as the “non-vehicle” class, but it is a vehicle. When this situation caused the accident, we can see it from the detailed result of running the image into the XAI model.
